# Combined transcriptomic and proteomic analysis reveals multiple pathways involved in self-pollen tube development and the potential roles of FviYABBY1 in self-incompatibility in *Fragaria viridis*

**DOI:** 10.3389/fpls.2022.927001

**Published:** 2022-09-14

**Authors:** Jianke Du, Chunfeng Ge, Tao Wang, Jing Wang, Zhiyou Ni, Shiwei Xiao, Fengli Zhao, Mizhen Zhao, Yushan Qiao

**Affiliations:** ^1^Laboratory of Fruit Crop Biotechnology, College of Horticulture, Nanjing Agricultural University, Nanjing, China; ^2^Institute of Horticulture Research, Zhejiang Academy of Agricultural Sciences, Hangzhou, China; ^3^Institute of Botany, Jiangsu Province and Chinese Academy of Sciences, Nanjing, China; ^4^Jiangsu Key Laboratory for Horticultural Crop Genetic Improvement, Institute of Pomology, Jiangsu Academy of Agricultural Sciences, Nanjing, China

**Keywords:** self-incompatibility, FviYABBY1, transcriptome, proteome, *Fragaria viridis*

## Abstract

*Fragaria viridis* exhibits *S*-RNase-based gametophytic self-incompatibility, in which *S*-RNase is the major factor inhibiting pollen tube growth. However, the pathways involved in and the immediate causes of the inhibition of pollen tube growth remain unknown. Here, interactive RNA sequencing and proteome analysis revealed changes in the transcriptomic and proteomic profiles of *F. viridis* styles harvested at 0 and 24 h after self-pollination. A total of 2,181 differentially expressed genes and 200 differentially abundant proteins were identified during the pollen development stage of self-pollination. Differentially expressed genes and differentially abundant proteins associated with self-incompatible pollination were further mined, and multiple pathways were found to be involved. Interestingly, the expression pattern of the transcription factor FviYABBY1, which is linked to polar growth, differed from those of other genes within the same family. Specifically, *FviYABBY1* expression was extremely high in pollen, and its expression trend in self-pollinated styles was consistent with that of *S-RNase*. Furthermore, FviYABBY1 interacted with *S*-RNase in a non-S haplotype way. Therefore, FviYABBY1 affects the expression of polar growth-related genes in self-pollen tubes and is positively regulated by *S*-RNase.

## Introduction

Self-incompatibility (SI) is a widespread mating system that avoids inbreeding and promotes outcrossing in angiosperms (Allen and Hiscock, 2008; Vieira et al., 2019; Kumar et al., 2021). In the family Rosaceae, the mechanisms of *S*-RNase-based gametophytic self-incompatibility (GSI) are diverse (Franklin-Tong and Franklin, 2003; McClure and Franklin-Tong, 2006; Sassa et al., 2010; Wu et al., 2013). In particular, studies on self- and non-self-recognition genera belonging, respectively, to tribes Amygdaleae (e.g., *Prunus*) and Maleae (e.g., *Malus* and *Pyrus*) have offered key insights into SI mating systems in the subfamily Amygdaloideae (Kubo et al., 2010; Iwano and Takayama, 2012; Fujii et al., 2016; Chen et al., 2020; Muñoz-Sanz et al., 2020; Harkness and Brandvain, 2021; Vekemans and Castric, 2021). In addition, an increasing number of modifiers have been explored, enabling comprehensive analyses of mechanisms underlying *S*-RNase-based SI (Wu et al., 2013; Claessen et al., 2019; Muñoz-Sanz et al., 2020). Within the genus *Fragaria* of the subfamily Rosoideae (family Rosaceae), some diploid species, such as *Fragaria viridis, F. nubicola, F. pentaphylla*, and *F. nipponica*, exhibit self-incompatibility as the major pollination barrier (Evans and Jones, 1967; Hancock, 1999; Sargent et al., 2004; Staudt, 2009; Bošković et al., 2010). The germplasm of low-ploidy wild strawberries contains abundant genetic resources controlling valuable traits, and it is a potential resource for improving cultivated strawberries (Hancock, 1999; Marta et al., 2004; Sargent et al., 2004; Staudt, 2009; Liston et al., 2014). However, as a pollination barrier between styles and pollen, SI has considerably hampered strawberry breeding (Evans and Jones, 1967; Li et al., 2000; Marta et al., 2004; Sargent et al., 2004; Bošković et al., 2010; Liston et al., 2014). As such, SI greatly limits the exploitation of valuable traits in diploid *Fragaria* species, such as enhanced disease resistance, stress tolerance, aromaticity, and solid content, for breeding (Evans, 1982; Hancock and Luby, 1993; Maas et al., 1997; Hancock et al., 2002; Ulrich et al., 2007). Therefore, elucidating molecular mechanisms underlying SI is expected to aid the development of strategies aimed at improving molecular breeding in strawberries. The mechanisms of SI in *Fragaria* have been studied for decades (Evans and Jones, 1967; Bošković et al., 2010; Aguiar et al., 2015). Specifically, GSI in *Fragaria* is known to be mediated by *S*-RNase (Du et al., 2021), similar to that in *Rosa* (Vieira et al., 2021); however, molecular mechanisms underlying the *S*-RNase-mediated inhibition of self-pollen tubes remain unclear.

High-throughput sequencing technologies, such as RNA sequencing (RNA-Seq) and proteomic analysis, have been used as efficient tools for studying SI mechanisms. In *Petunia inflate*, pollens of haplotypes *S*_2_ and *S*_3_ were analyzed using *de novo* transcriptome sequencing, and 17 pollen *SLF* determinants were identified (Williams et al., 2014; Wu et al., 2020). Moreover, comparative transcriptomics among unpollinated, self-pollinated, and cross-pollinated pistils of “Xiangshui” lemon revealed a series of genes related to pollen tube growth, programmed cell death (PCD), signal transduction, and transcription. In addition, a novel putative self-pollen rejection-associated *S*-RNase was detected, building the foundation for further research on SI mechanisms and the breeding of seedless varieties in lemons (Zhang et al., 2015). Additionally, proteomic analysis, as a tool for transcriptomics, has been widely applied in studies exploring SI mechanisms. Specifically, seven *S*-RNase-associated proteins have been identified in Japanese pears using two-dimensional gel electrophoresis (2-DE) (Ishimizu et al., 1996). Similarly, using 2-DE, Wang et al. (2013) discovered 22 proteins, some of which may be related to pollen maturation, fertility, germination, growth, and cell death. In a study exploring SI mechanisms in *Solanum pennellii*, proteomic analysis with isobaric tags for relative and absolute quantitation (iTRAQ) revealed that *S*-RNase, HT-A, cell wall-loosening proteins, and defense response-related proteins play key roles in an interspecific reproductive barrier between wild and domesticated species (Chalivendra et al., 2012). Overall, these cutting-edge techniques can not only directly identify potential pollen or style S-determinants but also reveal large-scale changes at the transcriptomic and proteomic levels in various SI-related biological processes during pollination.

Through ABCF transport proteins, *S*-RNase enters the pollen tube and functions non-specifically (Meng et al., 2014a,b). *S*-RNase is cytotoxic to self-pollen tubes, while its cytotoxicity is attenuated in non-self-pollen tubes (Claessen et al., 2019). Initially, the cytotoxicity of *S*-RNase was attributed to RNA degradation, and *S*-RNase was believed to enter the self-pollen tube and degrade RNA, explaining *S*-RNase-based GSI (Franklin-Tong and Franklin, 2003; Kubo et al., 2010; Tao and Iezzoni, 2010; Wu et al., 2013). *S*-RNase can break the gradient of reactive oxygen species (ROS) and calcium ions at the tip of the pollen tube, inducing a series of physiological and biochemical changes and PCD responses. Multiple signal transduction cascades are involved in *S*-RNase-based GSI (Wang et al., 2009, 2010; Wu et al., 2013, 2021; Li et al., 2018; Chen et al., 2020). Based on the evidence, the cytotoxicity of *S*-RNase in self-pollen tubes can be attributed to a multitude of reactions and signals, and the *S*-RNase-mediated degradation of RNA in pollen tubes may be the result of SI response or RNA homeostasis. The pollen tube is a classic model of polar growth, and the polarity of the self-pollen tube breaks following SI (Feijó, 2010; Wu et al., 2013; Del Duca et al., 2014). The *YABBY* gene family belongs to the zinc finger superfamily and plays pivotal roles in the development of vegetative and reproductive tissues in angiosperms (Kumaran et al., 2002; Bartholmes et al., 2012; Finet et al., 2016; Filyushin et al., 2017). As transcription factors, an important function of *YABBY*s is related to the establishment of polarity (Kumaran et al., 2002; Stahle et al., 2009); however, their link to SI and their involvement in breaking the polar growth of incompatible pollen tubes remain unknown.

The green strawberry *F. viridis* is a self-incompatible diploid (2n = 2x = 14) species. The fruits have an apple-like aroma, and the plants exhibit potent cold hardness and high soil pH tolerance (Labokas and Bagdonaitë, 2005; Staudt, 2009; Gruner et al., 2017). *F. viridis* is the germplasm for strawberry breeding (Gruner et al., 2017), and this species is a suitable model for further research on SI in the genus *Fragaria* (Bošković et al., 2010). In our experiment, at 24 h after self-pollination, most of the *F. viridis* pollen tubes stopped growing at 2/3 of the style. Thus, the receptacle with gynoecium from *F. viridis* line #10-42 (SI) at 0 and 24 h after self-pollination was selected for subsequent proteome sequencing. Integrative analysis with previous RNA-Seq data provided valuable information on the development of self-pollen tubes in the styles of *F. viridis*. In addition, the interaction between FviYABBY1 and *S*-RNase in *F. viridis* implicated polar growth regulators in self-incompatibility.

## Materials and methods

### Observation of pollen tube growth

Styles of *F. viridis* were self-pollinated and harvested at 1, 6, 12, 18, 24, and 48 h after pollination (HAP). The styles were fixed in FAA solution (5% formalin, 5% acetic acid, and 65% ethanol) for 2 h at 65°C. Next, the styles were washed with distilled water three times, treated with 4 M NaOH for 2 h at 65°C, and washed with distilled water three times before staining with 0.1% (w/v) aniline blue for 12 h at 4°C in the dark. Self-pollinated pistils of *Fragaria vesca* after 24 h were used as controls. The stained styles of *F. viridis* were placed on a glass slide and mounted in a drop of glycerol for observation under a fluorescence microscope (BX53; Olympus, Tokyo, Japan) at a wavelength of 356 μm.

### Transcriptomic data annotation

The self-pollinated receptacles with gynoecium from *F. viridis* lines #10–42 were harvested after 0 and 24 h and stored in liquid nitrogen for transcriptome sequencing and data analysis. Total RNA extraction, cDNA preparation, Illumina sequencing, and *de novo* assembly were performed as previously described (Du et al., 2021). The obtained non-redundant unigenes were annotated against the Nr, Nt, Swiss-Prot, COG, and KEGG databases using BLAST (Altschul et al., 1990), GO annotation was performed using Blast2GO, and InterPro annotation was performed using InterProScan5. ESTScan (Iseli et al., 1999) was used to predict the directions of unigenes that could not be annotated.

### Differential gene expression analysis

Unigene expression was estimated as fragments per kilobase of transcript per million fragments mapped reads (FPKM) (Mortazavi et al., 2008). Differentially expressed genes (DEGs) were detected using DEseq2 (Love et al., 2014), and *p-*values related to DEGs were modulated for multiple testing using the Benjamini–Hochberg false discovery rate (FDR) method (Benjamini and Hochberg, 1995). Unigenes deemed differently expressed were screened with an FDR-modulated *p-*value of ≤ 0.05 and |log_2_ FC| of ≥1. DEGs were subjected to GO functional and KEGG pathway analyses.

### Protein extraction, digestion, iTRAQ labeling, and strong cation exchange

iTRAQ analysis was performed at the Beijing Genomics Institute (BGI, Shenzhen, China). Plant material was the same as that for the RNA-Seq analysis described earlier. Two biological replicates were set per sample. Proteins were extracted using the trichloroacetic acid (TCA)/acetone method (Gu et al., 2013). Protein concentration was measured using the Bradford method. For each sample, 100 μg protein was digested with Trypsin Gold (Promega, Madison, WI, USA) at a protein-to-trypsin ratio of 20:1 for 4 h at 37°C. Following trypsin digestion, the peptides were vacuum centrifuged to dryness and dissolved in 0.5 M TEAB. iTRAQ labeling of peptide samples was performed using the iTRAQ^®^ Reagents-8plex Applications Kit according to the manufacturer's protocol. After labeling, the samples were combined and lyophilized, and the fractions were then analyzed using LC-ESI-MS/MS.

### Protein identification and quantification

MS/MS raw data were converted to MGF files using the ProteoWizard tool (msConvert, http://proteowizard.sourceforge.net), and proteins were searched using the Mascot search engine (Matrix, Science, London, UK, vision2.3.02) against the *de novo* transcriptomic data of *F. viridis* style (Du et al., 2021). Proteins were quantified using IQuant (BGI, Shenzhen, China), as previously described (Wen et al., 2014). Permutation tests were used for statistical and protein quantification analyses. Proteins were identified and quantified separately for each biological replicate (Tolin et al., 2013). FDR correction was adopted with a threshold of <1% to reduce the false identification of peptides. Proteins with a |log_2_ FC| of ≥0.6 and a *p*-value of ≤0.05 were considered differentially expressed.

### Analysis of YABBY family members in *F. viridis* and *F. vesca*

According to the hidden Markov model (HMM) of the YABBY domain, the *hmmsearch* program in HMMER (Finn et al., 2011) was used to search the whole-genome protein database of *F. viridis* and *F. vesca* for obtaining the *YABBY* gene family members. The selected proteins were input into the CDD database on NCBI (https://www.ncbi.nlm.nih.gov/cdd/?term) to verify the integrity of the YABBY domain, and proteins that did not contain the YABBY domain were filtered out. YABBY HMM (PF04690) can be downloaded directly from the Pfam database (http://pfam.xfam.org/). The protein database for *F. viridis* was derived and predicted based on the style transcript database after self-pollination (Du et al., 2021). The proteome database of *F. vesca* was downloaded from the Rosaceae Genome Database (https://www.rosaceae.org/), and *F. vesca* v4.0. a1 was selected.

Molecular weights and isoelectric points (pI) of *YABBY* gene family members were analyzed using ExPaSy (https://web.expasy.org/protparam/) (Artimo et al., 2012), and signal peptides were analyzed using SignalP (http://www.cbs.dtu.dk/services/SignalP/index.php) (Armenteros et al., 2019). The amino acid sequences of genes and proteins were compared using the alignment tool DNAMAN8.0 (Lynnon, Quebec, Canada). The online tool MEME (https://meme-suite.org/meme/tools/meme) was used for the motif analysis of YABBY family members, and TBtools was used for visualization. Using *Arabidopsis* and tomato as references (Bowman, 2000; Huang et al., 2013), multiple sequence alignment of YABBY protein sequences between *F. viridis* and *F. vesca* was performed with the MUSCLE program in MEGA 7.0 (Mello et al., 2017).

### Real-time quantitative PCR (RT-qPCR) validation of DEGs and *YABBY* family genes

Receptacles with gynoecium from *F. viridis* were collected at 0, 6, 12, 24, 48, and 72 HAP. Total RNA extraction and cDNA synthesis were performed as previously described (Du et al., 2021). Based on reference unigene sequences, 21 primer pairs (Supplementary Table S1) were designed using an online real-time PCR tool (https://sg.idtdna.com/scitools/Applications/RealTimePCR/). EF-1α was used as the internal control (Du et al., 2021). In addition, for spatiotemporal expression analysis, pollen, leaves, peduncles, calyxes, and petals of *F. viridis* were collected for tissue-specific expression analysis of *YABBY* family genes. The SYBR Premix Ex Taq™ kit (TaKaRa) was used for RT-qPCR. The reactions were conducted with three biological replicates using the ABI 7300 Real-Time System (PE Applied Biosystems, CA, USA). The 2^−ΔΔCT^ algorithm was used to analyze quantitative variation.

### Cloning of *FviYABBY1* in *F. viridis*

Primers were designed based on the open reading frame of the putative *FviYABBY1* unigene (Unigene16298_All) (Du et al., 2021) (see Supplementary Table S1). rTaq was used for PCR. The reaction conditions were as follows: 94°C for 4 min; 35 cycles of 94°C for 30 s, 55°C for 30 s, and 72°C for 1 min; and final extension at 72°C for 10 min. PCR products were evaluated on a 1% agarose gel. The target fragments were purified using the AxyPrep DNA gel extraction kit (Axygen, CA, USA), cloned into the pMD™ 19-T vector (Takara Bio, Dalian, China), and transformed into *Escherichia coli* DH5α competent cells. Positive clones were sequenced as described previously (Li et al., 2015).

### Subcellular localization assay

The *FviYABBY1* coding sequence (CDS) lacking stop codon was obtained from the plasmid containing *FviYABBY1* using primers with *Nco* I–*Spe* I restriction sites. *FviYABBY1* was ligated to the pCAMBIA1302-GFP expression vector using T4 ligase to construct the FviYAB1-GFP recombinant plasmid. Tobacco leaves were transiently transformed as described previously (Du et al., 2019). Following culture in the dark for ~60 h, the fluorescent signal of GFP in tobacco leaves was detected using a laser confocal fluorescence microscope.

### Yeast two-hybrid assay

The recombinant reporter vectors pGBKT7-Sa and pGBKT7-Sb were constructed, and yeast AH109 cells were transformed with the pGADT7 plasmid. To test the toxicity and self-activation of S_a_-RNase and S_b_-RNase, as well as to obtain the optimal 3-AT concentration for the background expression of eliminating reporter genes in yeast cells, pGADT7-largeT+pGBKT7-p53 and pGADT7-largeT+pGBKT7-laminC combinations were used as the positive and negative control, respectively. SD/-Trp/-Leu and SD/-Trp/-Leu/-His/Ade media with 3-AT concentration gradients of 0, 5, 10, and 15 mM were used, respectively.

The full-length CDSs of *S*_*a*_*-RNase* and *S*_*b*_-*RNase* were stored in the previously described vector as templates (Du et al., 2021), and primers with *Nde* I–*Bam* HI restriction sites were used to obtain the CDSs of mature S_a_-RNase and S_b_-RNase peptides. Enzyme digestion was used to construct the recombinant vectors pGBKT7-Sa and pGBKT7-Sb. The same method was used to recombine the full-length CDS of *F. viridis YABBY1* into pGADT7 for obtaining pGADT7-FviYAB1 with *Nde* I–*Bam* HI restriction sites. The primers used are listed in Supplementary Table S1.

The recombinant plasmids pGBKT7-Sa (pGBKT7-Sb) and pGADT7-FviYAB1 were co-transformed into the yeast strain AH109. The yeast cells were spread on the SD/-Trp-Leu medium and cultured for 3–5 days at 28°C in an incubator. Single colonies were selected, transferred to SD/-Trp-Leu liquid medium, and shaken. After shaking, the yeast suspension was straked on SD/-Trp-Leu, SD/-Trp-Leu-His-Ade, and SD/-Trp-Leu-His-Ade/X-α-gal plates and cultivated at 28°C in an incubator for 3–5 days to observe colony growth. pGADT7-largeT+pGBKT7-p53 and pGADT7-largeT+pGBKT7-laminC were used as the positive and negative controls, respectively.

### Validation of protein interaction using bimolecular fluorescence complementation (BiFC)

The target genes *FviYABBY1* and *S-RNase* (*S*_*a*_-RNase and *S*_*b*_-RNase) from the pMD19 (Simple) T-vector containing the corresponding gene were amplified using primers with restriction sites for the gene-specific primers used (Supplementary Table S1). Using restriction digestion, the CDS of *FviYABBY1* lacking the stop codon was cloned into the 35S:YCE vector with *Asc* I–*Bam* HI, and the full-length CDS of *S*_*a*_-RNase (*S*_*b*_-RNase) was cloned into the 35S:YNE vector with *Asc* I–*Kpn* I to obtain the recombinant vectors FviYABBY1-35S:YCE and Sa(Sb)-35S:YNE, respectively. Tobacco cells were transiently transfected with *Agrobacterium* following the same method as that for subcellular localization assay.

## Results

### Self-pollen tube growth in pistils

**Figure 1:**
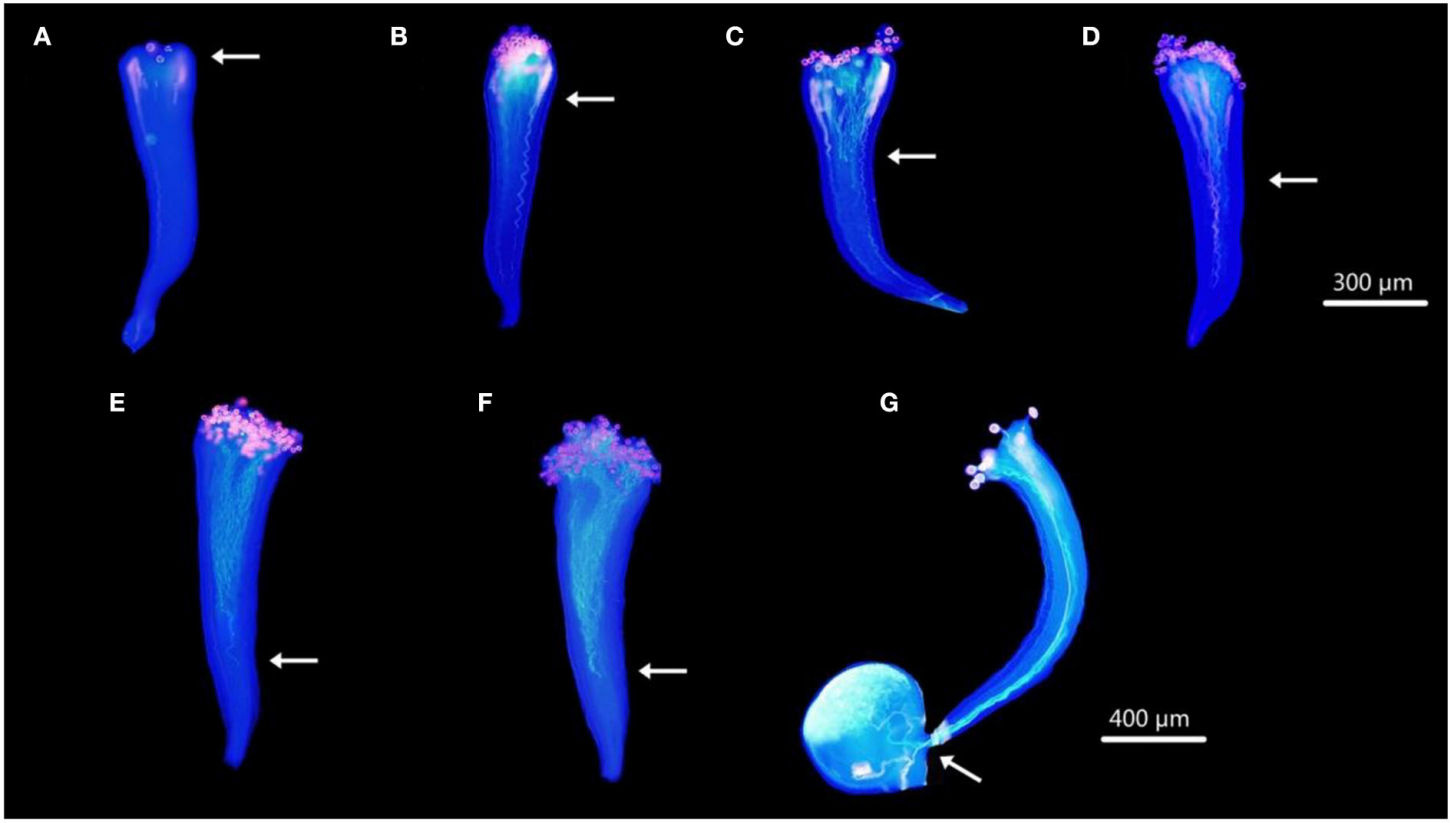
Dynamic development of pollen tubes in pistils under SP. **(A–F)** Styles of *F. viridis* at 1, 6, 12, 18, 24, and 48 HAP, respectively. **(G)** Pistil of *F. vesca* at 24 HAP. White arrows indicate the positions at which the majority of the pollen tubes were arrested in pistils. Bars **(A–F)** = 300 μm, **(G)** = 400 μm.

Aniline blue staining showed the growth of *F. viridis* pollen tubes in pistils at 1, 6, 12, 18, 24, and 48 HAP (Figures 1A–F). Pollen grains gradually attached to stigma papilla cells at 1 HAP, started germinating at 6 HAP (Figure 1B), and continued to elongate until reaching two-thirds of the pistil at 24 HAP (Figure 1E) refer to self compatible *F. vesca* (Figure 1G); no further distinct growth was observed at 48 HAP (Figure 1F). Thus, genes related to the inhibition of self-pollen tube growth may be expressed around 24 HAP based on the differential expression of pollen tube development. Accordingly, self-pollinated receptacles with gynoecia were harvested after 0 and 24 h and analyzed for differential expression.

### Transcriptomic analysis of styles at 0 and 24 h after self-pollination

**Figure 2:**
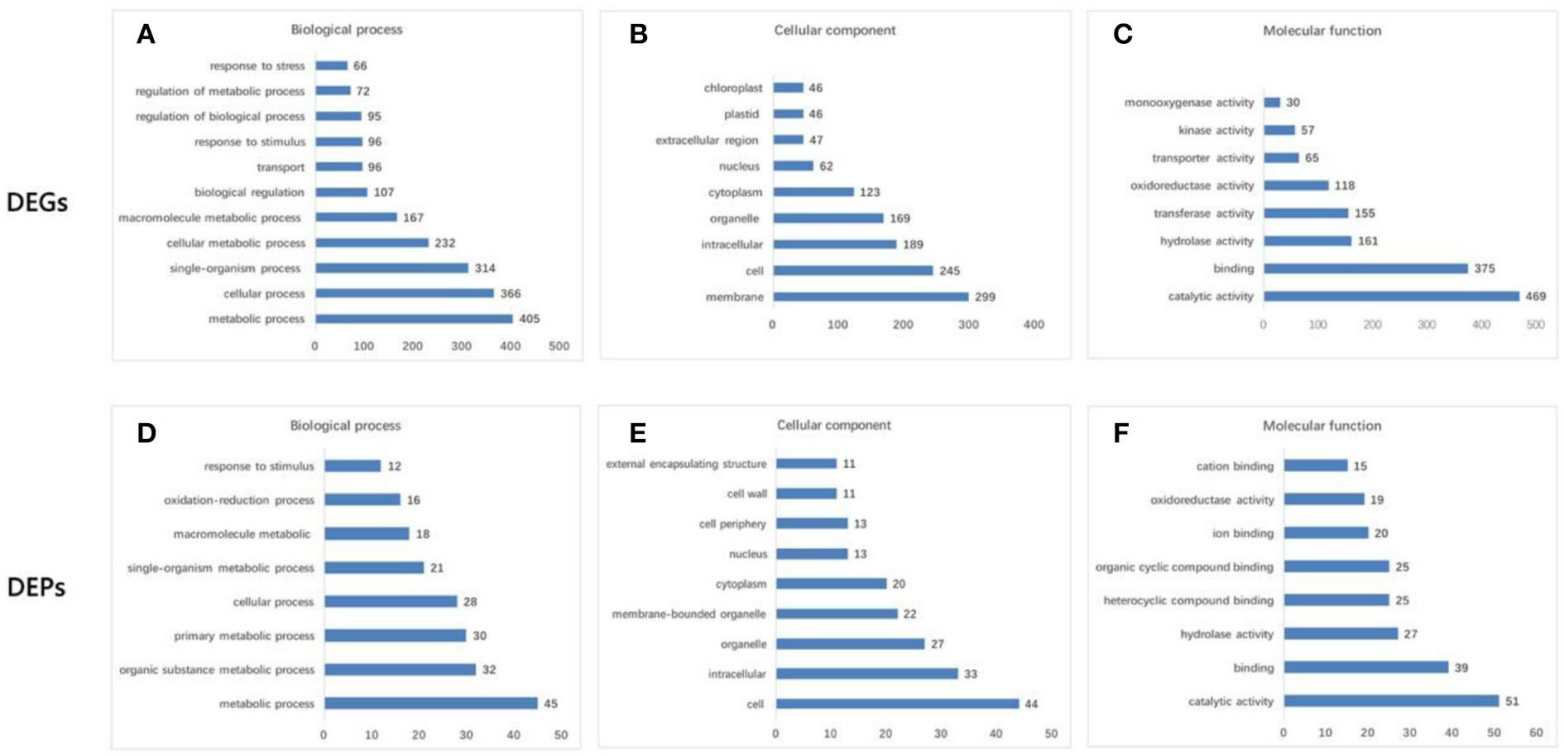
GO functional analysis of differentially expressed genes (DEGs) and differentially abundant proteins (DAPs). **(A,D)** Biological process. **(B,E)** Cellular component. **(C,F)** Molecular function. DEGs were mainly enriched in “metabolic process” (405 DEGs) and “cellular process” (366) for the biological process; “membrane” (299) and “cell” (245) for the cellular component; and “catalytic activity” (469) and “binding” (375) for the molecular function. The transcriptome data were significantly enriched in several GO categories, such as “cell wall organization and biogenesis,” “defense response,” “response to hormone,” “gametophyte development,” “pollen development,” “pollen germination,” and “pollination,” which are pollen–style interaction-associated biological processes. Moreover, GO terms annotation of DAPs revealed that “cell” (44) and “intracellular” (33) were the major cellular components; “catalytic activity” (51) and “binding” (39) were the major molecular functions, and “metabolic process” (45) and “cellular process” (28) were the major biological processes.

Six pools generated a total of 40.23 Gb of raw data (Supplementary Table S2). After removing low-quality, adaptor-polluted, and high-content unknown base (N) reads, 268.16 Mb of clean reads were generated (Q30 > 87.14%). A total of 71,756 unigenes were annotated using the Nr, Nt, Swiss-Prot, COG, KEGG, GO, and Interpro databases (Supplementary Figure S1A), with a mean length of 1,209 bp. The identified unigenes of *F. viridis* were highly homologous to those of *F. vesca* (85.5%), *Prunus mume* (1.59%), *Prunus persica* (1.31%), and *Malus domestica* (1.01%) (Supplementary Figure S1B, respectively). Global gene expression profiling between samples collected after 0 and 24 h revealed 2,181 DEGs, including 1,355 downregulated and 826 upregulated genes (Supplementary Table S3). These results indicate that many biological and molecular processes are altered during self-pollination. DEGs identified in the NR database were classified using GO functional analysis (Figures 2A–C), which is an important reference for pollen tube development in self-pollinated *Fragaria* species according to previous reports (Zhao et al., 2015; Zhang et al., 2016, 2017).

Furthermore, KEGG pathway analysis was performed to elucidate the biological pathways activated by pollen–style interaction. A total of 1,571 DEGs were annotated in 125 KEGG pathways (Supplementary Table S4). “Metabolic pathway” (ko01100), “biosynthesis of secondary metabolites” (ko01100), and “starch and sucrose metabolism” (ko00500) were the three major pathways. These results are consistent with the findings of transcriptome analysis of SI and SC pollination in tomatoes (Vieira et al., 2008). Interestingly, many genes were annotated to “plant–pathogen interaction” (ko04626), “phenylpropanoid biosynthesis” (ko00940), “plant hormone signal transduction” (ko04075), and “ubiquitin-mediated proteolysis” (ko04120), which are all notable pathways related to self-pollination (Elleman and Dickinson, 1999; Williams et al., 2015).

### Validation of gene expression using RT-qPCR

**Figure 3:**
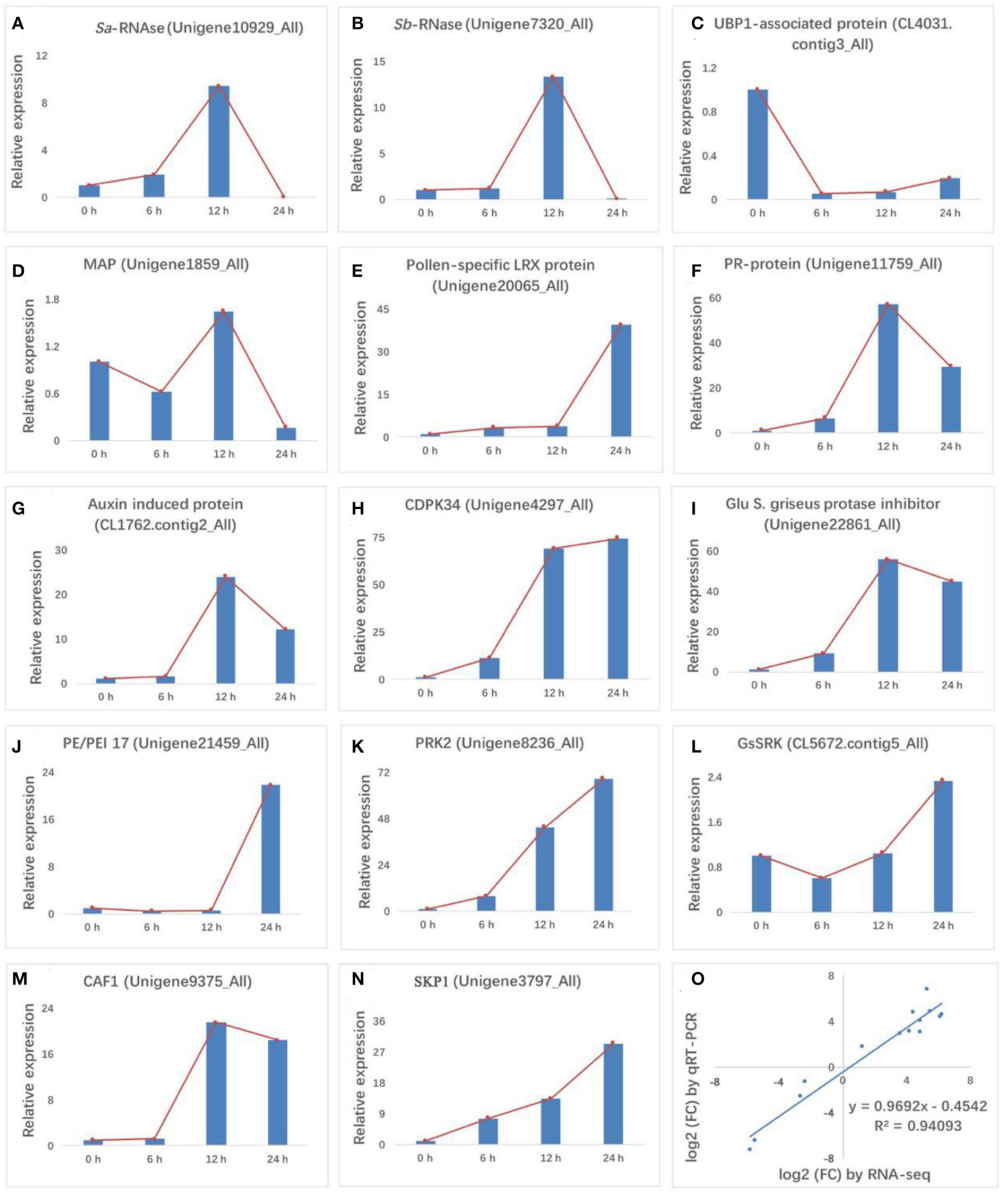
RT-qPCR validation of 14 differentially expressed genes (DEGs). **(A,B)** *S-RNase*, **(C)** cell death, **(D)** microtubule binding, **(E)** pathogen response, **(F,G)** hormone signaling, **(H)** CDPK signaling, **(I)** enzyme inhibitor activity, **(J)** cell wall, **(K,L)** pollen development, **(M)** RNA degradation, and **(N)** ubiquitin-mediated proteolysis were validated for gene expression levels at 0, 6, 12, and 24 HAP. **(O)** Comparison of fold-changes in gene expression (24 HAP/0 HAP) between RNA-Seq (x-axis) and RT-qPCR (y-axis) data. Both data were log_2_-transformed. In charts, red lines in **(A–N)** indicate the expression trend of each gene at the four pollination stages.

The quality of transcriptomic data was confirmed using RT-qPCR. Fourteen genes related to *S-RNase*s, cell death, microtubule binding, pathogen interaction, hormone signaling, CDPK signaling, enzyme inhibitor activity, cell wall, pollen development, RNA degradation, and ubiquitin-mediated proteolysis were detected (Figures 3A–N). The results showed good concordance (R^2^ = 0.9409) between the RT-qPCR and RNA-Seq data (Figure 3O), confirming the reliability of our transcriptomic data. Consistent with previous reports, *S*_*a*_-*RNase* and *S*_*b*_-*RNase* showed an initial uptrend, followed by a peak at 12 HAP and then a downtrend. Interestingly, the expression trends of four genes encoding a PR protein, an auxin-induced protein, a Glu S.griseus protease inhibitor, and CAF1 were similar to that of *S-RNase*. The expression levels of these four genes (Pollen-specific LRX protein, CDPK34, PRK2, and SKP1) increased after self-pollination. Conversely, the expression levels of three genes (UBP1 associated protein, PE/PEI 17, GsSRK) first decreased and then increased.

### Proteomic analysis and transcriptome–proteome integrative matching

Comparative proteomic analysis between the two samples collected at 0 and 24 HAP was performed as a parallel complement to transcriptomic analysis. We identified 7,105 proteins from 385,662 spectra (data not shown), including 200 DAPs (FC > 1.5, *p* < 0.05), of which 109 were upregulated and 91 were downregulated (Supplementary Table S5). According to the transcriptomic data, the results of GO functional analysis of DAPs and DEGs were highly consistent during self-pollination (Figure 2). In addition, 151 DAPs were annotated to 74 KEGG pathways (Supplementary Table S6), and the majority of these pathways were related to pollen tube development.

**Figure 4:**
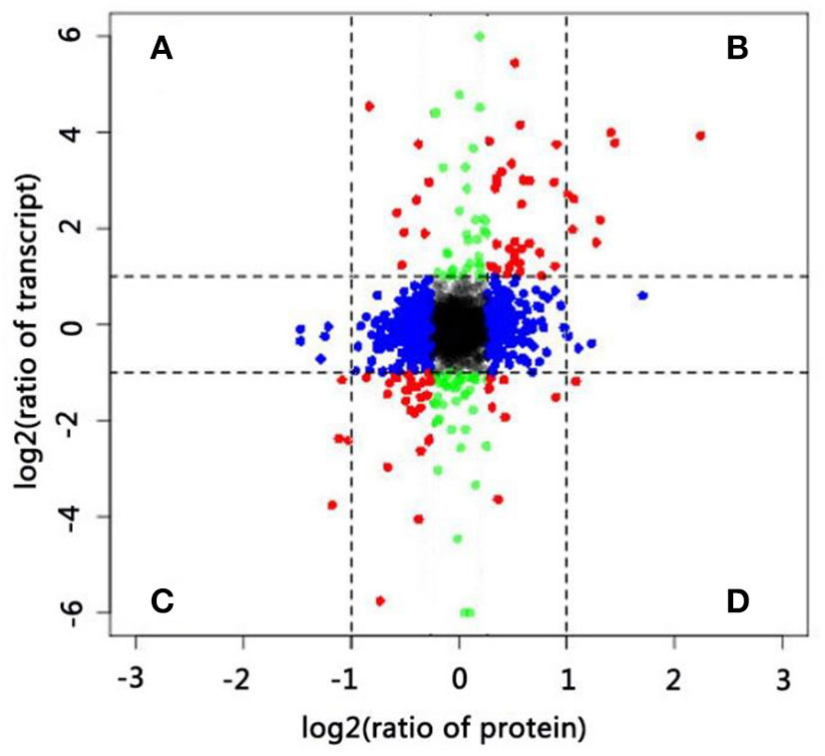
Correlations between protein and transcript levels. **(A–D)** Differentially expressed genes at both transcript and protein levels.

Integrative analysis of DEGs and DAPs offered comprehensive information on key genes involved in pollen–style interactions. In the present study, we identified 3,273 genes (Supplementary Table S7) at both transcript and protein levels and used these for correlation analysis. The distributions of the corresponding transcript-to-protein ratios (colored plots) were presented as scatter plots of log_2_-transformed data (Figure 4). The expression of most genes (black, blue, and green plots) did not significantly vary at the transcript (|log_2_ FC| ≥ 1) and protein (|log_2_ FC| ≥ 0.6) levels. Next, we focused on genes (red plot) in four sections (A–D). Genes in sections B and C exhibited similar expression trends at the transcript and protein levels (both upregulated and downregulated), whereas genes in sections A and D showed negative expression trends. Twelve DAPs (|log_2_ FC| ≥ 1 and *p* ≤ 0.05) that were differentially expressed at the transcript level (|log_2_ FC| ≥ 1 and *p* ≤ 0.05) are listed in Supplementary Table S8. Eleven genes showed consistent trends at both levels, including eight upregulated and three downregulated genes. Only one gene (beta-glucosidase 3) showed a negative expression trend, being downregulated at the transcript level but upregulated at the protein level at 24 HAP compared with that at 0 HAP. Beta-glucosidase is involved in various biological processes, including the formation and degradation of cell walls and response to biotic and abiotic stresses. The difference in the transcript and protein levels of this gene may result from many factors rather than its involvement in the self-incompatibility reaction alone.

### Data mining for DEGs and DAPs involved in SI

*S*-RNase-mediated GSI involves complex genetic mechanisms (Franklin-Tong and Franklin, 2003; Wu et al., 2013), and the integrity of SI depends on both the S locus (pistil S gene and pollen S gene) and the modifier genes (Sassa and Hirano, 2006; Wu et al., 2013; Claessen et al., 2019). These modifiers are closely related to PCD (Thomas and Franklin-Tong, 2004), plant-pathogen interactions (Elleman and Dickinson, 1999; Sanabria et al., 2008), Ca^2+^ signaling (Wu et al., 2013; Gu et al., 2015; Qu et al., 2016), ROS (Wang et al., 2010), hormone signaling (Shi et al., 2017), cell wall formation (Graaf et al., 2003), protein ubiquitination (McClure and Franklin-Tong, 2006), and pollen development (Zhang et al., 2016; Shi et al., 2017). Here, we clustered the SI-related DEGs and DAPs based on GO functional annotation, KEGG pathway classification, and Nr database annotation, as seen in Supplementary Table S9.

The pollen tube grows smoothly in a compatible style, which is regulated by apical polarity (Feijó, 2010; Del Duca et al., 2014). A gene encoding a transcription factor related to the polar growth of the pollen tube, the axial regulator *YABBY1* (Unigene16298_All), named *FviYABBY1*, was detected in the style at 0 HAP, and its expression was remarkably downregulated (log_2_ FC < −1) at 24 HAP. In the present study, *FviYABBY1* was annotated to a GO term but not to a protein or a KEGG pathway. The tip of self-incompatible pollen tubes cannot extend normally, which limits their polar growth (Franklin-Tong and Franklin, 2003; McClure and Franklin-Tong, 2006; Wu et al., 2013; Claessen et al., 2019). Therefore, we focused only on the gene related to the polar growth of pollen tubes and explored its link to self-incompatibility through the following series of experiments.

### Screening, basic characteristics, and homology analysis of *YABBY* genes in *F. viridis*

The *YABBY* gene family comprises plant-specific transcription factors playing vital roles in many biological processes during plant growth and development. These genes are differentially expressed after self-pollination. To gain a comprehensive understanding of their roles and homologs in SI, the *YABBY* family genes expressed in the style and pollen interaction system of *F. viridis* were screened using *F. vesca* as the control. Through screening and verification, 13 *YABBY* family members were finally obtained—seven and six from the style and pollen interaction system of *F. viridis* and the whole genome of *F. vesca*, respectively. These genes were named according to their homology with the *Arabidopsis YABBY* family genes (Bowman, 2000), and their basic characteristics were analyzed (Supplementary Table S10). The identified YABBY family members harbor two conserved domains—a C_2_C_2_ zinc finger structure and a YABBY domain—and the degree of amino acid conservation in the YABBY domain is greater than that in the zinc finger structure (Supplementary Figure S2). In addition to the conserved motifs, the various members of the YABBY family contain unique motifs (Supplementary Figure S3), implying that these genes share certain commonalities and functional specificities.

**Figure 5:**
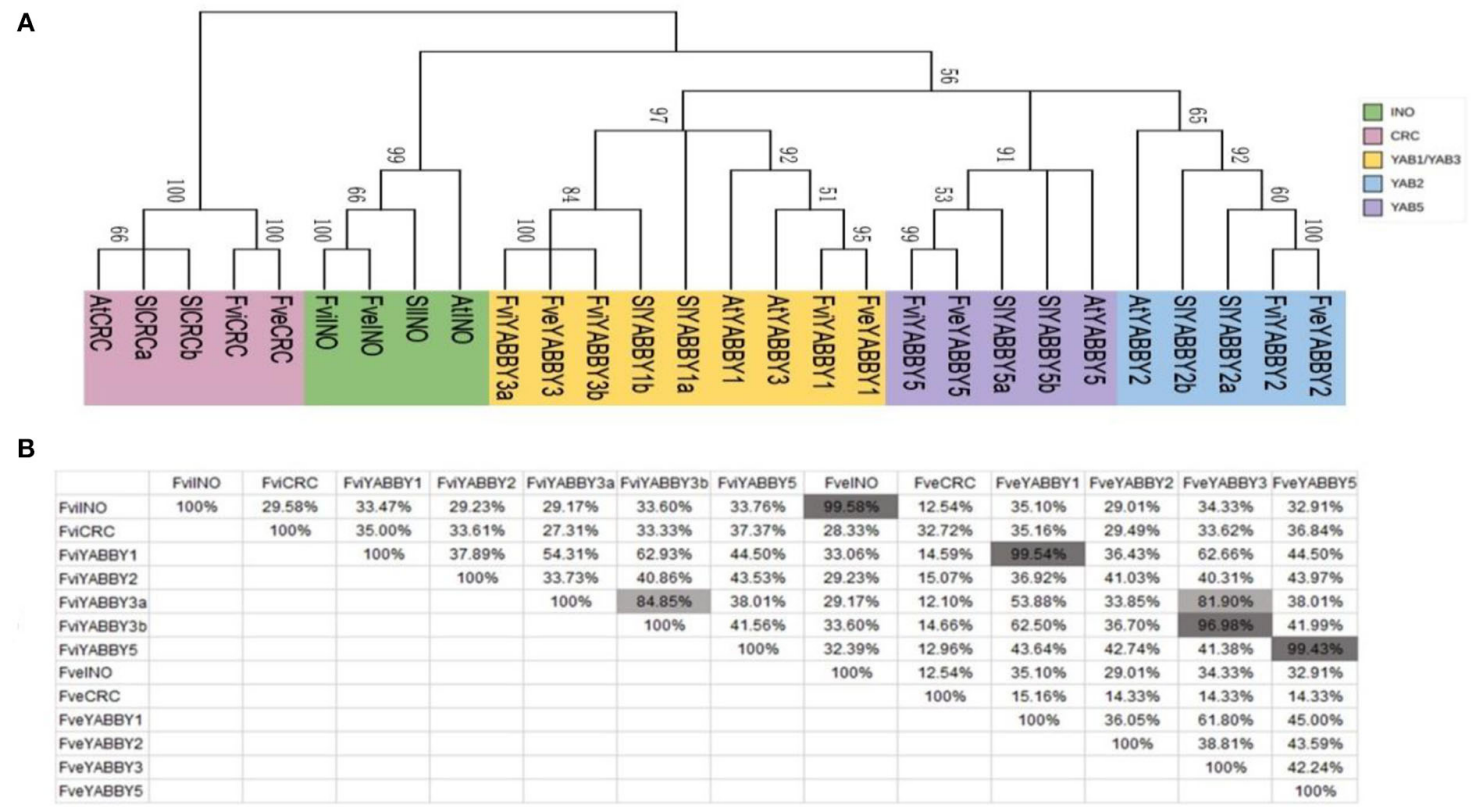
Homology analysis of YABBY family members in *Fragaria viridis* and *Fragaria vesca*. **(A)** Phylogenetic tree of YABBY family members in *F. viridis* and *F. vesca*. **(B)** Amino acid similarity between YABBY family members in *F. viridis* and *F. vesca*.

Consistent with previous reports in Arabidopsis, tomato, rice, wheat, and pomegranate (Bowman, 2000; Toriba et al., 2007; Huang et al., 2013; Zeeshan et al., 2020; Zhao et al., 2020), the *YABBY* genes in *F. viridis* and *F. vesca* were divided into five subfamilies: INO, CRC, YAB2, YAB1/YAB3, and YAB5 (Figure 5A). Similar to those in *F. vesca*, INO, CRC, YAB2, and YAB5 in *F. viridis* comprise one member, while the remaining subfamily YAB1/YAB3 comprises three members, including one in YAB1 and two in YAB3, respectively. Meanwhile, in *F. vesca, Arabidopsis* (Bowman, 2000), and tomato (Huang et al., 2013), YAB1/YAB3 comprises two members, one each in YAB1 and YAB3.

Homology analysis revealed that the functions of INO and YAB5 subfamily members are not differentiated between *F. viridis* and *F. vesca*, with similarity exceeding 99.43%; however, the YABBY2 and CRC subfamily members share a low similarity at 41.03 and 32.72%, respectively (Figure 5B). The YABBY1 and YABBY3 proteins in strawberries shared high homology, both within and between species, which explains the clustering of members in these two groups and their classification in the YAB1/3 subfamily. In addition, among the members of the strawberry YABBY family, only two YAB3 members (FviYABBY3a and FviYABBY3b) are present in *F. viridis*, both sharing high similarity, which suggests the occurrence of a gene duplication event in YAB3 throughout the evolution. The amino acid similarity was 99.54% between FviYABBY1 and FveYABBY1, 96.98% between FveYABBY3 and FviYABBY3a, and 81.90% between FveYABBY3 and FviYABBY3b, suggesting that FviYABBY3b has undergone functional diversification following the gene duplication event.

### Analysis of *YABBY* gene expression patterns in *F. viridis*

**Figure 6:**
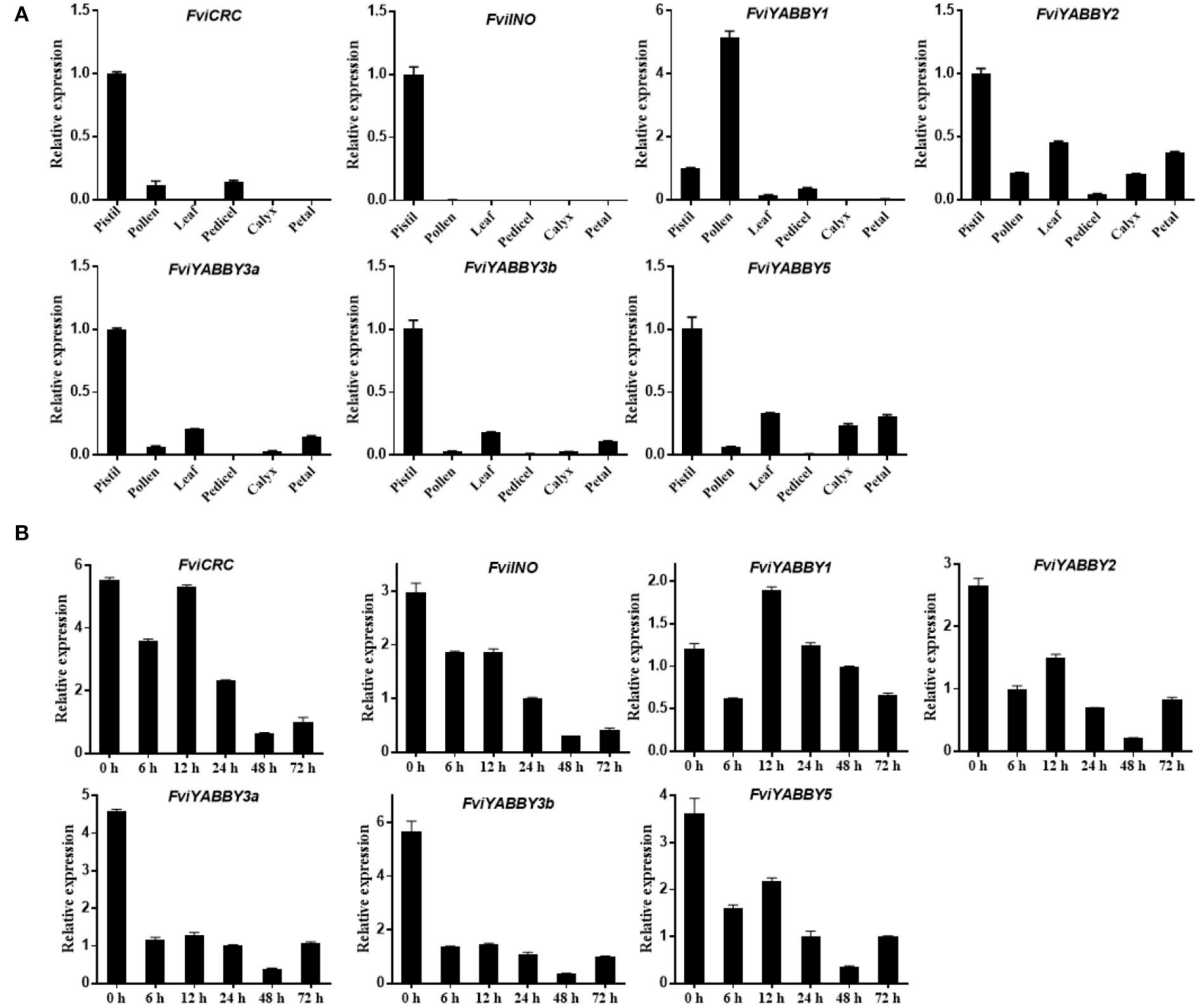
Analysis of gene expression pattern using RT-qPCR. **(A)** Tissue-specific expression patterns of *YABBY* family genes in *Fragaria viridis*. **(B)** Spatiotemporal expression after self-pollination of *YABBY* family genes in *F. viridis*.

The expression levels of *YABBY* family genes in the pistil, pollen, leaves, peduncle, calyx, and petals of *F. viridis* were analyzed using RT-qPCR. As seen in Figure 6A, the expression levels of *FviYABBY* genes were relatively high in the pistil, suggesting their important roles in pistil development. Unlike other family members, *FviYABBY1* showed the highest expression level in pollens; thus, the key function of FviYABBY1 may be in the pollen. We further analyzed the expression levels of these genes in pistils at different stages after self-pollination. As seen in Figure 6B, except for *FviYABBY1*, the other *YABBY* family genes reached their peak expression levels at 0 HAP. Further, while the expression of all *FviYABBY* genes significantly decreased at 6 h, their expression levels increased at 12 h, following another downward trend at 24 and 48 HAP. The YABBY family members were clustered on the phylogenetic tree and harbored the same conserved domains (Figure 5 and Supplementary Figure S3). *FviYABBY3a* and *FviYABBY3b* as well as *FviYABBY2* and *FviYABBY5* were highly similar in terms of their tissue-specific expression patterns and expression patterns in pistils at different stages after pollination, implying that they may serve similar functions. Although the expression level of *FviYABBY1* in pistils decreased at 6 HAP, it reached the peak value at 12 HAP and then declined again. The expression trend of *FviYABBY1* was consistent with that of *S-RNase* (Du et al., 2019), suggesting that *FviYABBY1* is positively regulated by *S*-RNase.

### *FviYABBY1* cloning and subcellular localization

**Figure 7:**
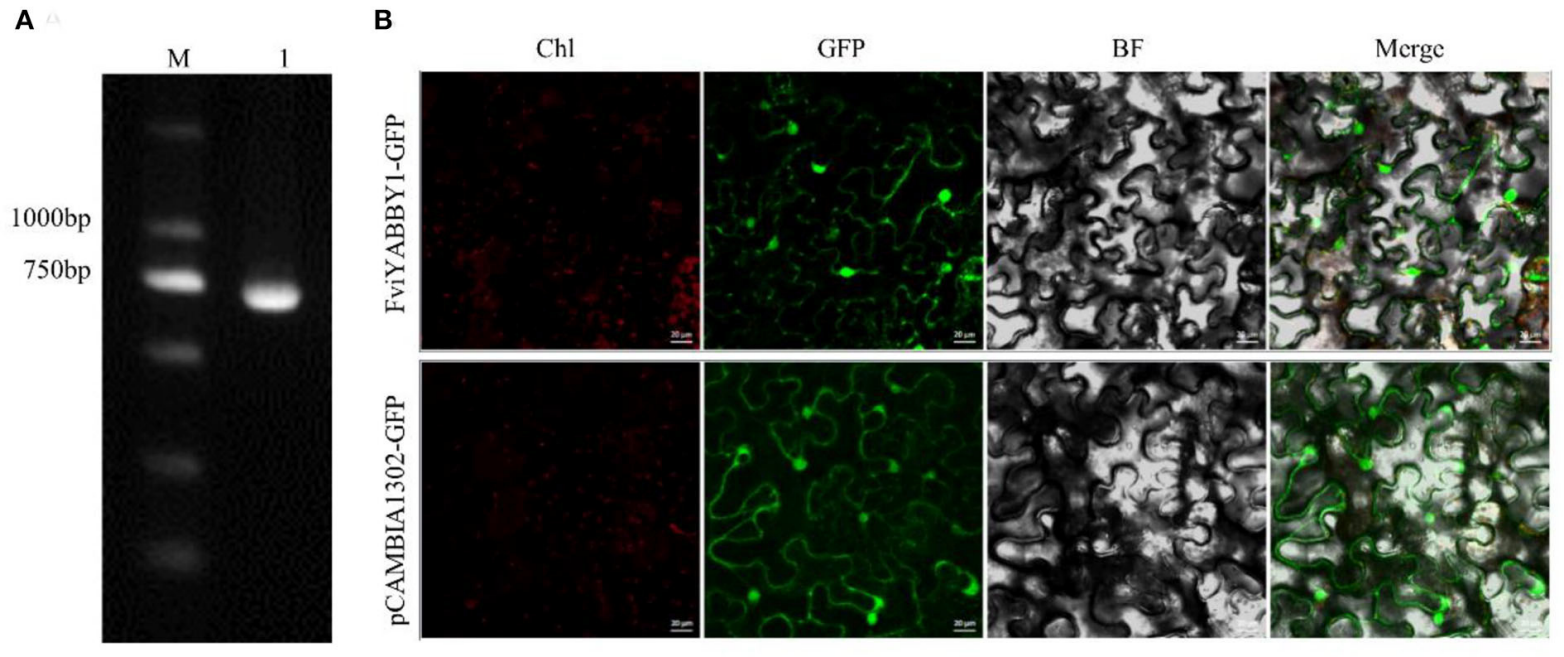
Gene cloning and subcellular localization of FviYABBY1. **(A)** *FviYABBY1* cloning. M: 2000 DNA Marker; 1: Fragment of *FviYABBY1* CDS. **(B)** Subcellular localization of FviYABBY1. Chl, chlorophyl II autofluorescence; GFP, green fluorescence protein; BF, bright field; Merge, merged image of Chl, GFP, and BF. Bar = 20 μm.

Using *F. viridis* pistil cDNA as the template, the CDS-specific target band was obtained by PCR amplification (Figure 7A), with a length of 657 bp. Monoclonal sequencing results were consistent with the transcriptome data (Du et al., 2021). *FviYABBY1* was cloned into pCAMBIA1302-GFP, and the recombinant vector was transferred into tobacco leaves through *Agrobacterium*-mediated transformation. The cellular distribution of FviYABBY1 protein was determined based on the GFP signal. Under 488 nm excitation wavelength, green fluorescent signals were observed in the nucleus and the cytoplasm (Figure 7B). Therefore, FviYABBY1 lacks a signal peptide or transmembrane structure (Supplementary Table S10) and is a non-secreted protein that cannot be transferred between cells.

### Interaction of FviYABBY1 with S_a_-RNase and S_b_-RNase

S_a_-RNase and S_b_-RNase are non-toxic and non-self-activated in yeast cells. Thus, yeast two-hybrid assays were conducted on SD/-Trp-Leu-His-Ade medium without 3-AT to verify whether other proteins interact with the target RNases (Supplementary Figure S4).

**Figure 8:**
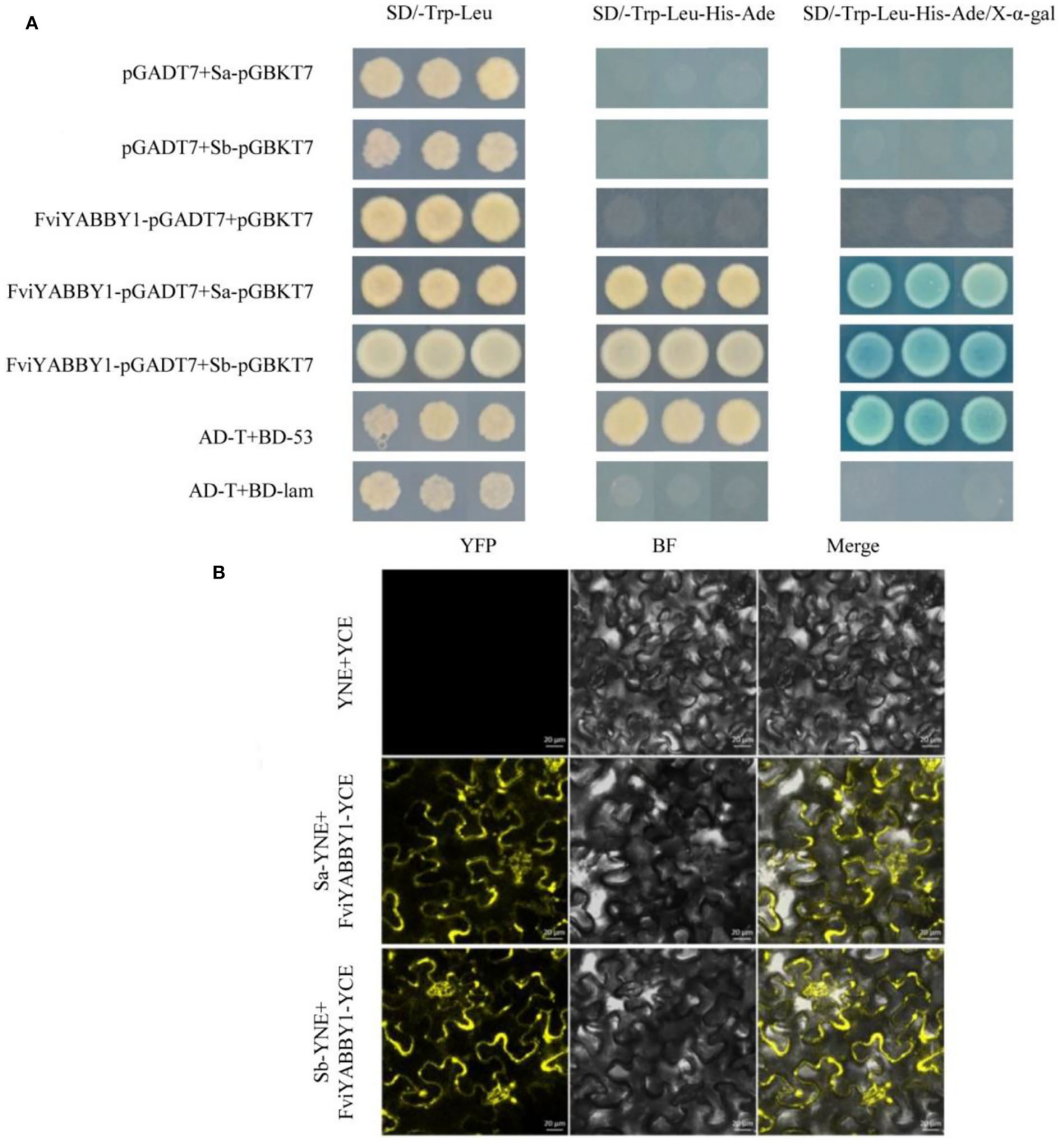
Interaction between FviYABBY1 and S_a_-RNase/S_b_-RNase. **(A)** Yeast two-hybrid assay. **(B)** Bimolecular fluorescence complementation (BiFC) assay. YFP, yellow fluorescence protein; BF, bright field; Merge, merged image of YFP and BF. Bar = 20 μm.

The recombinant plasmids FviYABBY1-pGADT7+S_a_-pGBKT7 and FviYABBY1-pGADT7+S_b_-pGBKT7 were co-transformed into the yeast strain AH109. The positive yeast strains on the SD/-Trp-Leu medium were multiplied and inoculated on prefabricated plates containing SD/-Trp-Leu, SD/-Ade-His-Trp-Leu, and SD/-Trp-Leu-His-Ade/X-α-gal media. As seen in Figure 8A, all yeast strains grew normally on SD/-Trp-Leu medium, indicating that yeast co-transformation was successful. Yeast strains containing FviYABBY1-pGADT7+S_a_-pGBKT7 grew stably on thr SD/-Ade-His-Trp-Leu medium, while those containing FviYABBY1-pGADT7+S_b_-pGBKT7 grew stably on the SD/-Trp-Leu-His-Ade medium and grew blue colonies on the SD/-Trp-Leu-His-Ade/X-α-gal medium. These results indicate the interaction of the FviYABBY1 protein with S_a_-RNase and S_b_-RNase without *S*-RNase haplotype specificity.

We further verified the protein interaction results of FviYABBY1 with S_a_-RNase and S_b_-RNase and the location of the interaction. The recombinant vectors were co-transferred into tobacco cells using *Agrobacterium* harboring FviYABBY1-35S:YCE+S_a_-35S:YNE and FviYABBY1-35S:YCE+S_b_-35S:YNE. As seen in Figure 8B, the tobacco cells containing FviYABBY1-35S:YCE+S_b_-35S:YNE and FviYABBY1-35S:YCE+S_b_-35S:YNE exhibited fluorescence, whereas control cells harboring YCE+35S:YNE did not. The results of the BiFC assay confirmed that FviYABBY1 interacts with S_a_-RNase/S_b_-RNase and that these interactions are not S-haplotype specific. Further, based on fluorescence signals, the cytoplasm is the main location of FviYABBY1–*S*-RNase interaction.

## Discussion

### *S*-RNase-based GSI in *F. viridis*

*Fragaria viridis* is a typical self-incompatible gametophyte. In this species, SI primarily manifests in the pollen tube, which is strongly inhibited in the styles (Evans and Jones, 1967). In a previous study, a pair of style determinants (S_a_-RNase and S_b_-RNase) was discovered based on transcriptomic data at 0 and 24 HAP, proving the occurrence of *S*-RNase-based GSI in *Fragaria* (Du et al., 2021). Consistent with previous reports, most self-incompatible pollen tubes eventually stop growing at 2/3 of the style in the genus *Fragaria* (Evans and Jones, 1967; Du et al., 2019, 2021). Contrary to that in other species, such as *Solanum* and *Pyrus*, the self-incompatible pollen tube in *F. viridis* showed a greater extension degree in styles, suggesting differences in the intermediate mechanisms or SI modifiers among self-incompatible species (Franklin-Tong and Franklin, 2003; Zhang et al., 2009; Baek et al., 2015; Claessen et al., 2019). Therefore, we further analyzed the previously obtained transcriptomic data and combined these with proteomic data to unveil the mechanisms of pollen tube development and pollen tube growth cessation due to SI in *F. viridis*. FviYABBY1, a putative transcription factor related to the polar growth of pollen tubes, has been a research hotspot, and its roles in *F. viridis* SI are discussed in this article.

### DEGs and DAPs related to SI

In *S*-RNase-based GSI systems, a type of pollen–pistil interaction, pollen grains germinate and grow normally on the stigma, and the rejection of self-pollen tube is mainly determined by the style (Franklin-Tong and Franklin, 2003; Hiscock and Allen, 2008; Shi et al., 2017; Seth et al., 2019; Aloisi et al., 2020). In the present study, the majority of the *F. viridis* pollen tubes were arrested in the style at 24 HAP (Figure 1E), implying that certain time-specific genes (24 HAP) regulating pollen tube growth are the key targets of the *Fragaria* SI system. Successful manifestation of SI entails the involvement of modifiers other than the *S-locus* determinants (McClure et al., 2000; Sassa and Hirano, 2006). Here, we identified 2,181 genes and 200 proteins that were differentially expressed between 0 and 24 HAP in receptacles with gynoecia, and many SI-related genes were further predicted and classified (Supplementary Table S9). Interestingly, many additional DEGs and DAPs were enriched in GO terms, such as “pollen development,” “recognition of pollen,” “pollen germination,” and “pollen–style interaction.” Arabinogalactan proteins (AGPs) and the AGP extension hybrid glycoprotein TTS are closely linked to pollen development and control pollen tube elongation during *S*-RNase-based GSI (Majewska-Sawka and Nothnagel, 2000; Seifert and Roberts, 2007). Furthermore, genes encoding GDSL esterase/lipase protein, fasciclin-like arabinogalactan protein, stigma-specific stig1-like protein, and glycosyltransferase are involved in pollen development and germination (Goubet et al., 2003; Updegraff et al., 2009; Zhang et al., 2022). In addition, pollen receptor-like kinase (RLK) is a key factor in pollen–style recognition (Escobar-Restrepo et al., 2007), and RALF-like proteins play critical roles in the regulation of pollen tube growth (Covey et al., 2010; Murphy and De Smet, 2014; Zhang et al., 2020). All these genes were classified into the category “pollen development” and most of them were significantly upregulated at 24 HAP (Supplementary Table S9). These results suggest that the expression of the above genes is closely related to self-pollen development in styles. Pollen is a typical model of cell polar growth. Thus, a polar growth regulator of DEGs was further investigated according to functional annotation. Although the potential functions of these genes in *Fragaria* SI require further confirmation, our results indicate that some regulators related to pollen growth are involved in the SI response of *F. viridis*.

SI is an intricate biological process closely associated with ubiquitin-mediated proteolysis, which eventually leads to PCD and RNA degradation (Thomas and Franklin-Tong, 2004; McClure and Franklin-Tong, 2006; Zhang et al., 2009). In the present study, we found that SKP1 (Unigene3797_All), as a component of the ubiquitin–ligase complex, was gradually upregulated after self-pollination (FC > 29) at 24 h compared with the expression level at 0 h in the validation data (Figure 3N). Two DAPs (CL4737_Contig1_All and CL7114_Contig_All) and one DEG (Unigene8982_All) were annotated as E3 ubiquitin-protein ligases. A series of PCD-related DEGs and DAPs, such as MAPKKKs, 18.2 kDa class I heat shock protein (Unigene11193_All), and cytochrome P450, were identified (Supplementary Table S9), all of which are important regulators of PCD according to previous reports (Ludovico et al., 2002; del Pozo et al., 2004; Li et al., 2012). As the activity of *S*-RNase is protected in self-pollen tubes, *S*-RNase degrades cytoplasmic RNA, arrests protein synthesis, and finally inhibits pollen tube growth (Kao and Tsukamoto, 2004; Wang et al., 2009; Zhang et al., 2015). In the present study, 15 DEGs and 2 DAPs were enriched in the “RNA degradation” pathway (Supplementary Tables S4, S6), including one CCR4-associated factor (CAF), four NAC domain-containing proteins, three chaperonin 60 subunit beta 4, and two uncharacterized proteins (Supplementary Table S9). Many of these genes were annotated to “RNA degradation” in self-pollination for the first time in this work, and further research may reveal their necessity in the pollen–style interaction pathways.

Biological processes, such as “cell wall modification,” “accumulation extracellular calcium,” “synthesis of β-1,3 glucan callose,” and “presence of phenolic derivatives,” are common between SI and pathogen resistance (Zhou et al., 2014). In addition, enzymes, such as pectin lyase, cellulases, and glucanases, are typically secreted by pathogens for cell wall degradation (Wing et al., 1990; Yoder et al., 1990; Ham et al., 1997; Aich et al., 2017). Similar to that during pathogen defense (Wing et al., 1990; Marín-Rodríguez et al., 2002), cell wall modifiers, such as pectate lyase 5, beta-glucosidase 3, and glucan endo-1,3-beta-glucosidase, were downregulated at the transcript and protein levels (Supplementary Table S9). Ca^2+^ is vital in pathogen interactions, and the transient influx of Ca^2+^ across the plasma membrane plays a major role during the early stages of pathogen response (Zimmermann et al., 1997). In our transcriptome data, several Ca^2+^ signaling-related genes were markedly upregulated; as such, these genes were enriched in the KEGG pathway “plant–pathogen interaction” (Supplementary Figure S5 and Supplementary Table S9). As for pathogen response, hormones are essential for incompatibility response (Graaf et al., 2003; Robert-Seilaniantz et al., 2007; López et al., 2008; Song et al., 2013; Zhang et al., 2016). Here, we detected 56 DEGs and 8 DAPs that were enriched in the KEGG pathway “plant hormone signal transduction” (Supplementary Tables S4, S6). These findings support that the mechanism of plant–pathogen interactions are remarkably similar to that of pollen tube development and pollen–style recognition (Hodgkin et al., 1988; Thomas and Franklin-Tong, 2004).

### Functional role of *YABBY* genes in *F. viridis* SI

*YABBY* family genes are seed-plant-specific transcription factors with critical roles in vegetative and reproductive development (Kumaran et al., 2002; Bartholmes et al., 2012; Finet et al., 2016; Filyushin et al., 2017; di Rienzo et al., 2021; Romanova et al., 2021; Luo et al., 2022). *S*-RNase-based SI occurs when pollen tubes stop growing and polar growth is hindered in self-styles (Wu et al., 2013; Claessen et al., 2019). Using transcriptomic data as clues, we focused on *YABBY* family genes, particularly *YABBY1*, which may be involved in the regulation of pollen tube polar growth. YABBY1 is primarily expressed in pollen and lacks a signal peptide, suggesting that it functions in the pollen. Yeast two-hybrid and BiFC assays confirmed that YABBY1 interacts with *S*-RNase, albeit without haplotype specificity. In species exhibiting SI, *S*-RNase enters the pollen tube non-specifically (Meng et al., 2014a,b) and exerts a toxic effect in the self-pollen tube; meanwhile, the toxicity of non-self *S*-RNase is eliminated (Wu et al., 2013; Sassa, 2016; Claessen et al., 2019). Therefore, self-*S*-RNase likely interacts with YABBY1 in the pollen tube, transmits the *S*-RNase cytotoxicity signal, and affects the polar growth of the pollen tube. In the non-self-pollen tube, the cytotoxicity of *S*-RNase is relieved by pollen determinants. In self-compatible *F. vesca*, given the loss of the *S-RNase* gene, *YABBY1* executes the normal function of regulating the polar growth of the pollen tube tip. *YABBY1* is positively correlated with *S-RNase* in an incompatible style–pollen interaction system after self-pollination. Therefore, *S*-RNase may regulate the expression of SI-related genes by positively regulating the expression of *YABBY1*.

SI is a system in which style interacts with pollen (Bedinger et al., 2017). In addition to *YABBY1*, six *YABBY* family members from *F. viridis* were highly expressed. Some *YABBY* family genes exhibited similar expression patterns and may be correlated to some extent. Genes in the same family may serve similar functions (Li et al., 2016; Lin et al., 2018); thus, other genes in the *YABBY* family may also be related to SI. However, whether they are involved in the SI response of *F. viridis*, similar to *YABBY1*, warrants further experimental exploration.

## Data availability statement

The datasets presented in this study can be found in online repositories. The names of the repository/repositories and accession number(s) can be found in the article/Supplementary materials.

## Author contributions

JD, CG, and TW co-performed the experiment design, the major experiment operation, data handling, and manuscript writing. JW, ZN, SX, and FZ performed partial experiments. YQ and MZ proposed the research idea and experimental design and assisted with manuscript editing. All authors contributed to the article and approved the submitted version.

## Funding

This work was supported by the National Natural Science Foundation of China (Nos. 32072540 and 31872056), the Fundamental Research Funds for the Central Universities (No. KYZZ2022004), Postgraduate Research & Practice Innovation Program of Jiangsu Province (No. KYCX21_0612), and the Jiangsu Province Agricultural Science and Technology Innovation Fund Projects [No. CX(21)2019].

## Conflict of interest

The authors declare that the research was conducted in the absence of any commercial or financial relationships that could be construed as a potential conflict of interest.

## Publisher's note

All claims expressed in this article are solely those of the authors and do not necessarily represent those of their affiliated organizations, or those of the publisher, the editors and the reviewers. Any product that may be evaluated in this article, or claim that may be made by its manufacturer, is not guaranteed or endorsed by the publisher.

## Abbreviations

DEG: differentially expressed gene
DAP: differentially abundant protein
FC: fold change
GSI: gametophytic self-incompatibility
SLF: *S-locus* F-box protein
PCD: programmed cell death
SI: self-incompatibility
SC: self-incompatibility
iTRAQ: isobaric tags for relative and absolute quantitation
Nt: NCBI nucleotide sequences
Nr: NCBI non-redundant protein
COG: Cluster of Ortholog Genes
GO: Gene Ontology
KEGG: Kyoto Encyclopedia of Genes and Genomes
RNA-Seq: RNA sequencing
FDR: false discovery rate
2-DE: two-dimensional gel electrophoresis
RT-qPCR: real-time quantitative PCR
FPKM: fragments per kilobase per million mapped reads
TCA: trichloroacetic acid
AGP: arabinogalactan proteins
CDPK: calcium-dependent protein kinase
ROS: reactive oxygen species.
