# Supplementary material for: Combined transcriptomic and proteomic analysis reveals multiple pathways involved in self-pollen tube development and the potential roles of FviYABBY1 in self-incompatibility in Fragaria viridis
Source: Front Plant Sci. 2022 Sep 14;13:927001. doi: 10.3389/fpls.2022.927001 (PMC9515988; doi:10.3389/fpls.2022.927001)
Supplement: Supplementary file 11 [file Data_Sheet_1.docx]

**Supplementary data**

**Supplementary Fig. S1.** Summary for transcriptome annotation. **A.** Venn diagram of transcripts annotated to five databases. **B.** gene distribution in highly homological species.

**Supplementary Fig. S2. Multiple sequences alignment of the YABBY family genes among *A. thaliana*, *F. vesca* and *F. viridis.*** **Note:** Conserved cysteine residues in the zinc-finger domain are indicated with asterisks.

**Supplementary Fig. S3. Phylogenetic and conserved domain analysis of the YABBY gene family in *F. viridis* and *F. vesca.*** **A.** Phylogenetic relationships of YABBY family in *F. viridis*; **B.** Conserved domain of YABBY family in *F. viridis*; **C.** Phylogenetic relationships of YABBY family in *F. vesca*; **D.** Conserved domain of YABBY family in *F. vesca*.

**Supplementary Fig. S4. Self-activation detection of Sa and Sb-RNase under different 3-AT concentrations.** **Note:** After the vectors was introduced into the AH109 yeast strain, and a single clone was selected for activating, diluting, and coating on SD/-Trp-Leu-His-Ade deficient plates with different 3-AT concentrations (0 mM, 5 mM, 10 mM and 15 mM), and cultured at 30 ^o^C for 3 days. combinations The positive control strain (containing pGADT7-largeT and pGBKT7-p53) can grow at a concentration of 0-15 mM 3-AT. However, the suppression phenomenon has been appeared, the growth area of single colony and the number of colonies gradually decrease with the increase of 3-AT concentration. Consistent with the negative control (pGADT7-largeT and pGBKT7-laminC), the Sa-RNase (Sb-RNase) bait recombinant vector together with the pGADT7 empty plasmid were introduced into the AH109 yeast, the strain can not grow on SD/-Trp-Leu-His-Ade plate. Growth (pGADT7 and pGBKT7-Sa; pGADT7 and pGBKT7-Sb). The yeast strains contain different vectors (pGADT7-largeT and pGBKT7-p53, pGADT7-largeT and pGBKT7-laminC, pGBKT7-Sa and pGADT7, pGBKT7-Sb and pGADT7, respectively) can grow normally on SD/-Trp-Leu culture medium, suggested that Sa and Sb-RNase had no effect on the normal growth of yeast.

**Supplementary Fig. S5. DEGs involved in plant-pathogen interaction KEGG pathway. Note:** Red box indicates DEGs were up-regulated, green box indicates DEGs were down-regulated, red and green colored boxes indicated both up-regulated and down-regulated DEGs were involved, black box indicates genes involved in the pathway did not significantly expressed.

**Supplementary Table S1.** Primers used for RT-qPCR and cloning.

**Supplementary Table S2.** Summary for de novo transcriptome.

**Supplementary Table S3.** DEGs between pistils harvested at 0 and 24 HAP. The significance level was set at p-value ≤ 0.05 and Fold change (FC) > 2. FC: Fold change (24 HAP / 0 HAP).

**Supplementary Table S4.** KEGG pathways of DEGs.

**Supplementary Table S5.** DAPs between pistils harvest at 0 and 24 HAP.

**Supplementary Table S6.** KEGG pathways of DAPs.

**Supplementary Table S7.** Genes used for correlation analysis at transcript and proteomic level.

**Supplementary Table S8.** Table S8. Genes were detected at both transcriptomic and proteomic levels.

**Supplementary Table S9.** Vital DEGs and DAPs identified by RNA-seq and iTRAQ during SP. FC: Fold change (24 HAP / 0 HAP).

**Supplementary Table S9.** Physicochemical properties of YABBY family genes in *F. viridis* and *F. vesca.*


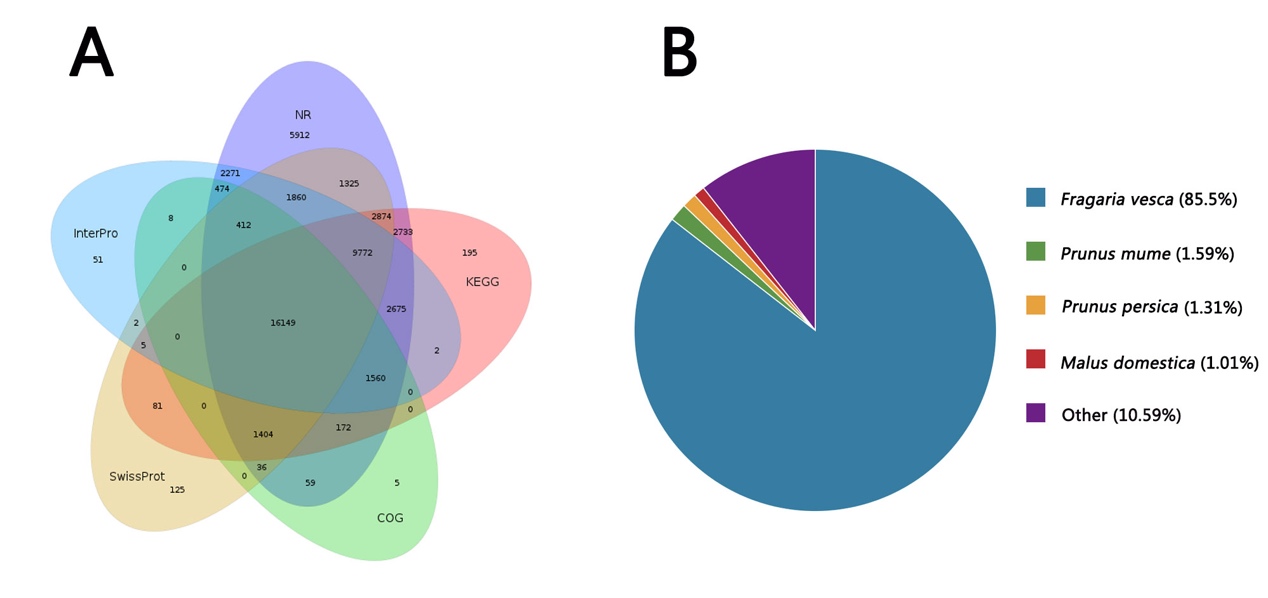


**Supplementary Fig. S1.** Summary for transcriptome annotation. **A.** Venn diagram of transcripts annotated to five databases. **B.** gene distribution in highly homological species.


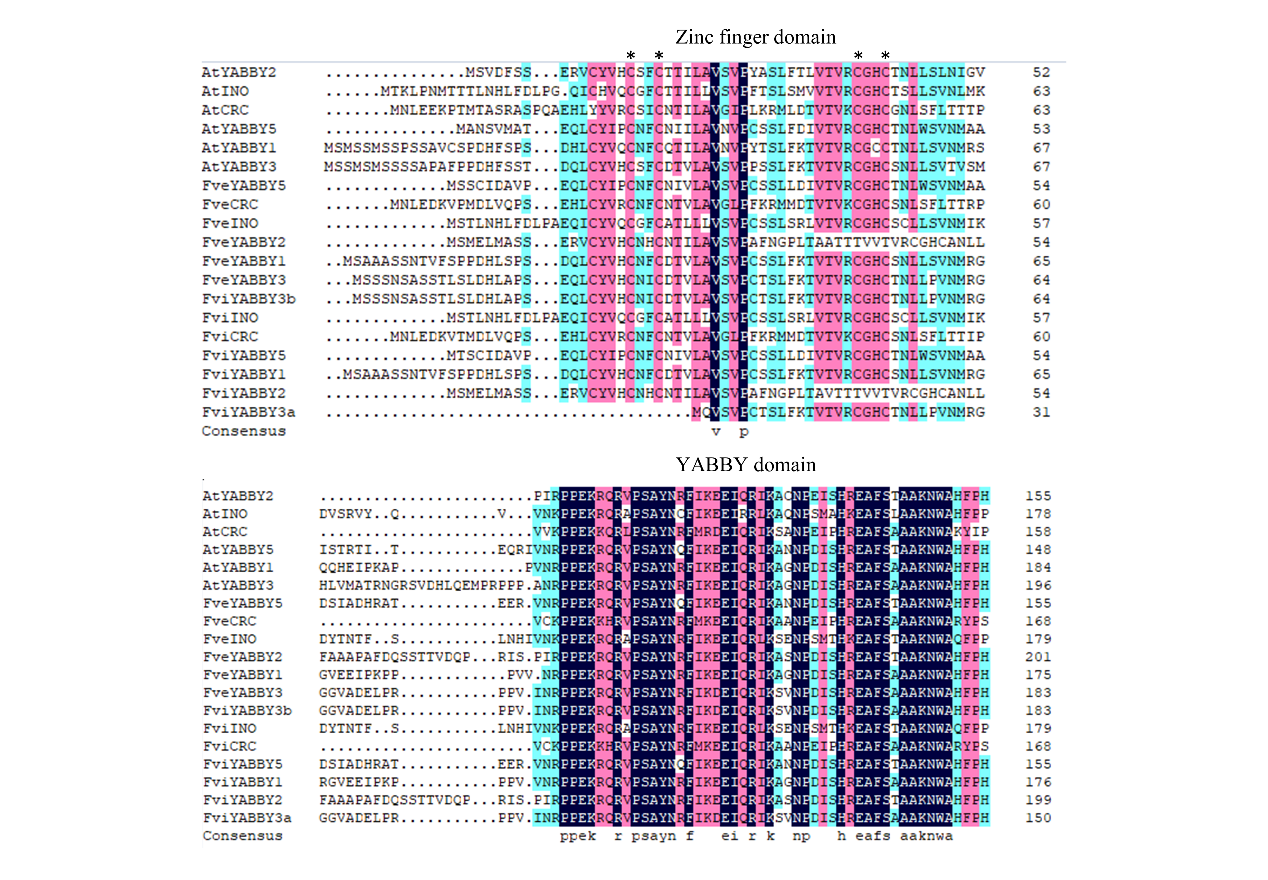


**Supplementary Fig. S2. Multiple sequences alignment of the YABBY family genes among *A. thaliana*, *F. vesca* and *F. viridis.*** **Note:** Conserved cysteine residues in the zinc-finger domain are indicated with asterisks.


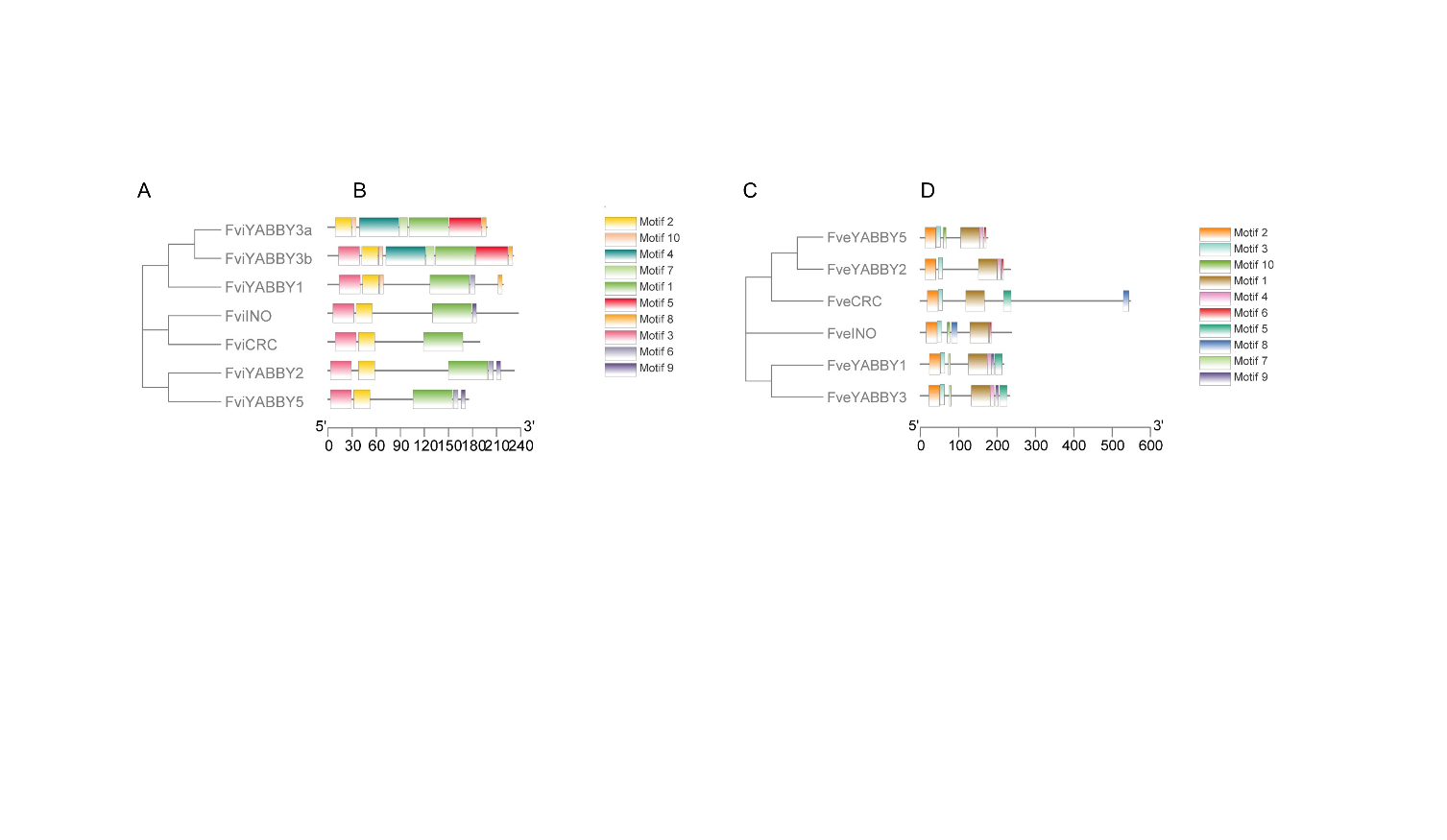


**Supplementary Fig. S3. Phylogenetic and conserved domain analysis of the YABBY gene family in *F. viridis* and *F. vesca.*** **A.** Phylogenetic relationships of YABBY family in *F. viridis*; **B.** Conserved domain of YABBY family in *F. viridis*; **C.** Phylogenetic relationships of YABBY family in *F. vesca*; **D.** Conserved domain of YABBY family in *F. vesca*.


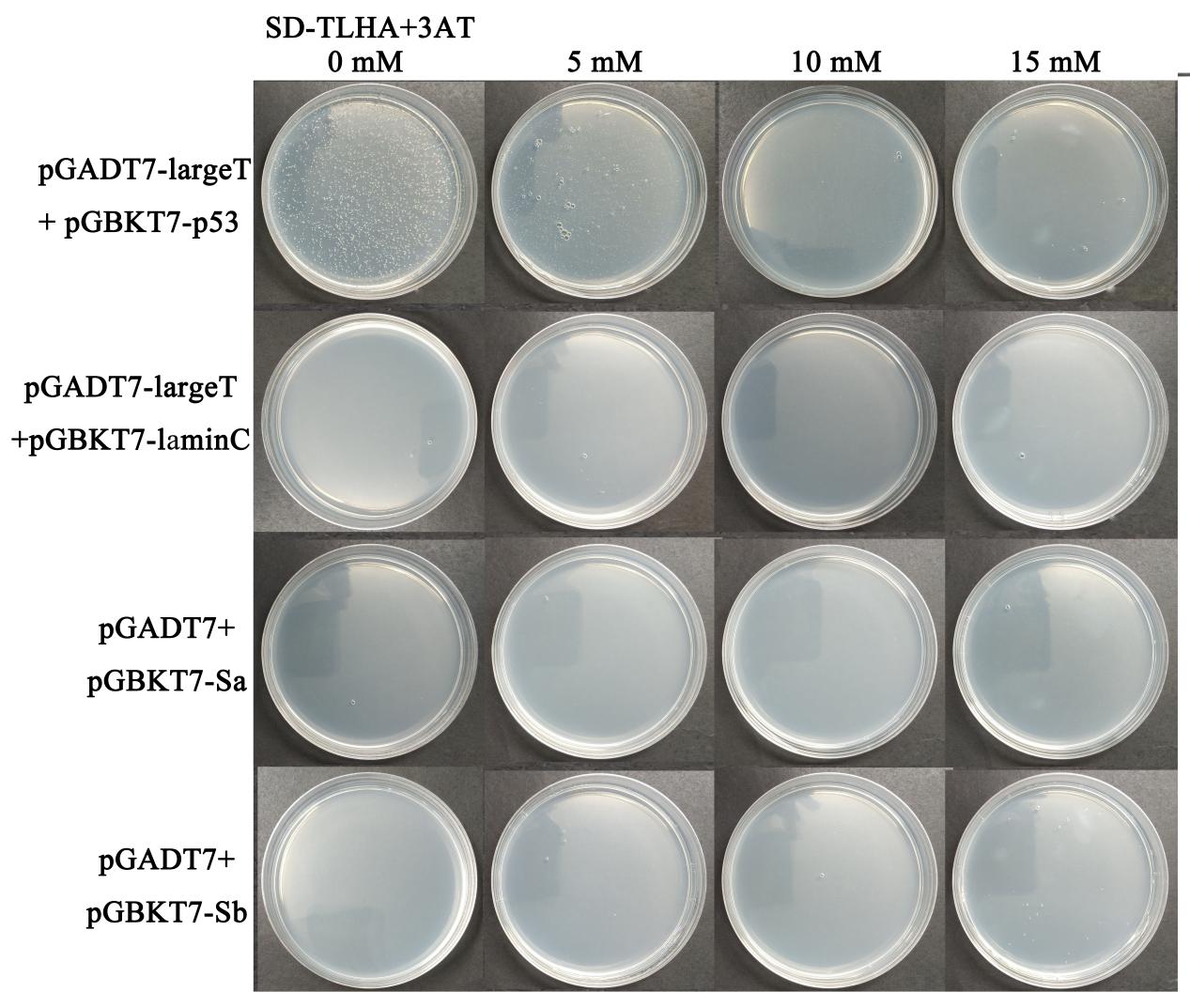


**Supplementary Fig. S4. Self-activation detection of Sa and Sb-RNase under different 3-AT concentrations.** **Note:** After the vectors was introduced into the AH109 yeast strain, and a single clone was selected for activating, diluting, and coating on SD/-Trp-Leu-His-Ade deficient plates with different 3-AT concentrations (0 mM, 5 mM, 10 mM and 15 mM), and cultured at 30 ^o^C for 3 days. combinations The positive control strain (containing pGADT7-largeT and pGBKT7-p53) can grow at a concentration of 0-15 mM 3-AT. However, the suppression phenomenon has been appeared, the growth area of single colony and the number of colonies gradually decrease with the increase of 3-AT concentration. Consistent with the negative control (pGADT7-largeT and pGBKT7-laminC), the Sa-RNase (Sb-RNase) bait recombinant vector together with the pGADT7 empty plasmid were introduced into the AH109 yeast, the strain can not grow on SD/-Trp-Leu-His-Ade plate. Growth (pGADT7 and pGBKT7-Sa; pGADT7 and pGBKT7-Sb). The yeast strains contain different vectors (pGADT7-largeT and pGBKT7-p53, pGADT7-largeT and pGBKT7-laminC, pGBKT7-Sa and pGADT7, pGBKT7-Sb and pGADT7, respectively) can grow normally on SD/-Trp-Leu culture medium, suggested that Sa and Sb-RNase had no effect on the normal growth of yeast.


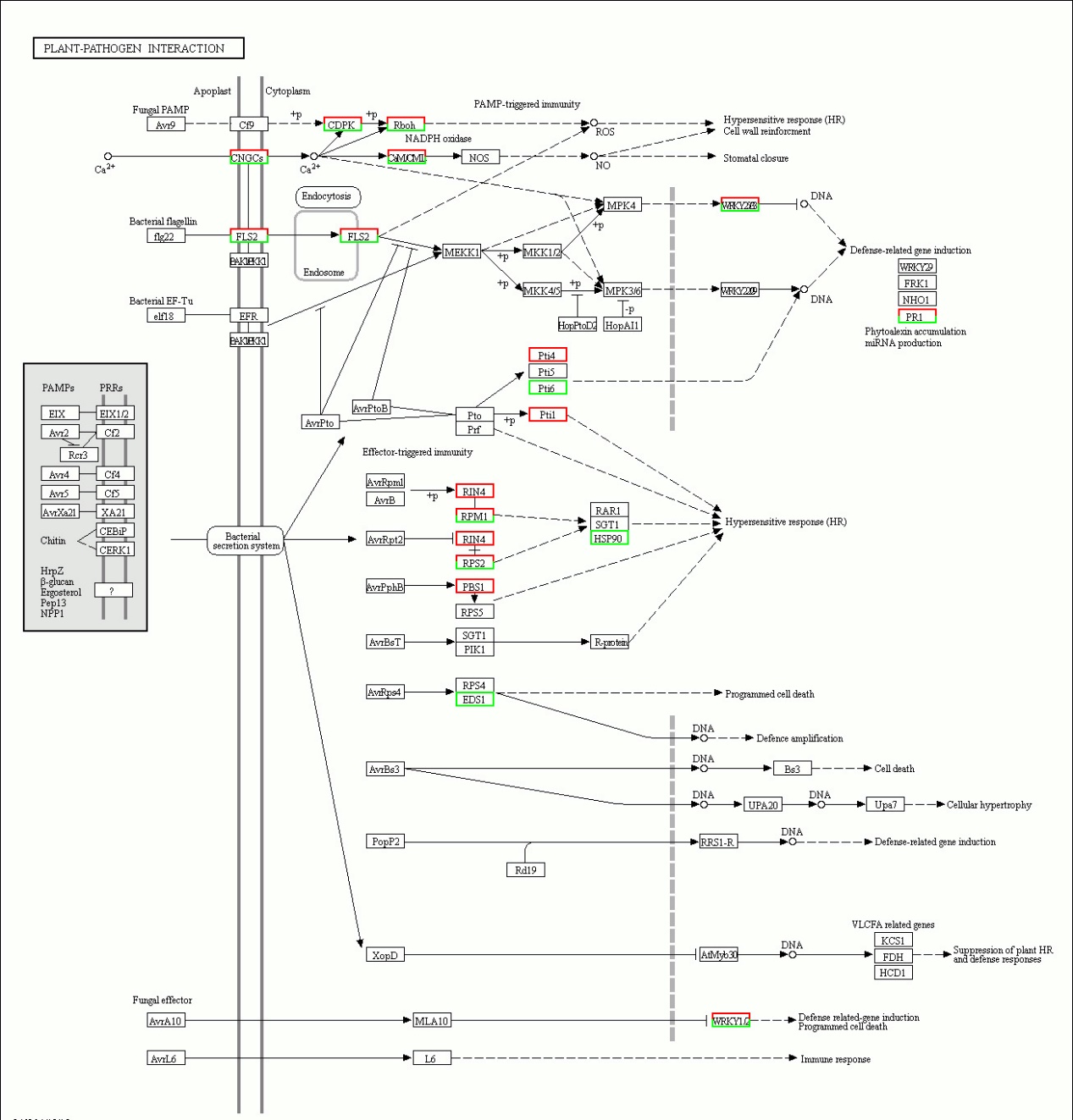


**Supplementary Fig. S5. DEGs involved in plant-pathogen interaction KEGG pathway. Note:** Red box indicates DEGs were up-regulated, green box indicates DEGs were down-regulated, red and green colored boxes indicated both up-regulated and down-regulated DEGs were involved, black box indicates genes involved in the pathway did not significantly expressed.
